# The signal transduction pathway of PKC/NF-κB/c-fos may be involved in the influence of high glucose on the cardiomyocytes of neonatal rats

**DOI:** 10.1186/1475-2840-8-8

**Published:** 2009-02-11

**Authors:** Wang Min, Zhang Wen Bin, Zhou Bin Quan, Zhu Jun Hui, Fu Guo Sheng

**Affiliations:** 1 The Department of Cardiovascular Diseases, Sir Run Run Shaw Hospital, College of Medicine, Zhejiang University, Hangzhou, Zhejiang Province, PR China

## Abstract

**Background:**

High glucose could induce structure and function change in cardiomyocytes, PKC plays a core effect in the onset and progression of diabetic cardiomyopathy, but its underlying downstream signal transduction pathway is still not completely understood.

**Objectives:**

To study the influence of high glucose on the structure, function and signal transduction pathway of PKC (Protein Kinase C)/NF-κB(Nuclear factor-κB)/c-fos in cultured cardiomyocytes.

**Methods:**

Using cultured cardiomyocytes of neonatal Sprague-Dawley rats as a model, groups were divided into: control group (glucose: 5 mmol/L); high glucose group (glucose: 10 mmol/L, 15 mmol/L, 20 mmol/L, 25.5 mmol/L); equimolar mannital group (5 mmol/L glucose + 20.5 mmol/L maninital); high glucose(25.5 mmol/L) add PKC inhibitor (Ro-31-8220, 50 nmol/L); high glucose (25.5 mmol/L) add NF-κB inhibitor (BAY11-7082, 5 μmol/L). The cellular contracting frequency and volumes were measured and the expression of PKC-α, PKC-β2, p-PKC-α, p-PKC-β2, NF-κB, p-NF-κB, TNF-α (tumor necrosis factor-α) and c-fos were measured by western blot or RT-PCR.

**Results:**

Cardiomyocytes cultured in high glucose level, but not iso-osmotic mannital, showed an increased pulsatile frequency and higher cellular volumes consistent with the increased glucose levels, and also higher expression of PKC-α, PKC-β2, p-PKC-α, p-PKC-β2, NF-κB, p-NF-κB, TNF-α and c-fos. The addition of Ro-31-8220 and BAY11-7082 could partly reverse these changes induced by high glucose level.

**Conclusion:**

High glucose significantly increased the pulsatile frequency and cellular volumes of cultured cardiomyocytes via PKC/NF-κB/c-fos pathway, which might lead to diabetic cardiomyopathy.

## Background

Diabetes mellitus is a state of chronic hyperglycemia due to an absolute or relative deficiency of insulin secretion that may or may not be associated with insulin resistance. The worldwide prevalence of diabetes was estimated to be 2.8% in 2000 and is projected to reach 4.4% by 2030[1]. Diabetic cardiomyopathy is one of the most prevalent cardiovascular complications of diabetes mellitus that occurs independently of coronary artery disease and hypertension[2]. Many epidemiological and clinical studies have shown that chronic hyperglycemia is a major initiator of diabetic microvascular and cardiovascular complications as high glucose may regulate the growth of cardiomyocytes via activates several signal transduction pathways[3]. For example, hyperglycemia could accelerate polyol pathway flux, alter cellular redox state, increase formation of diacylglycerol (DAG) and the subsequent activation of protein kinase C (PKC) isoforms and augmented non-enzymatic formation of advanced glycated end products, which cause the extracellular matrix to change and induce hypertrophy of cardiomyocytes, microangiopathy of heart, fibrosis of interstitial substance, which eventually leading to heart failure[4,5]. Among the signal pathways listed above, the DAG-PKC signal pathway is considered to be one of the most important intracellular transduction pathways that functions as a core effect in the onset and progression of diabetic cardiomyopathy.

Approximately more than 10 different isozymes make up the PKC family, with respect to the heart, PKC-α and PKC-β2 are the predominant Ca^2+^-dependent PKC isoforms[6]. A number of reports have associated PKC activation with many cardiovascular abnormalities in cardiomyopathy, as it affects cardiovascular function in many ways, such as cardiac hypertrophy, dilated cardiomyopathy, ischemic injury[7,8]. Studies have revealed that increased DAG levels and PKC activity in diabetic cardiomyopathy are associated with changes in blood flow, thickening in basement membrane, expansion of extracellular matrix, increasing in vascular permeability and abnormality of angiogenesis. Also increased expression and activity of PKC can lead to excessive cardiomyocyte apoptosis and alteration of enzymatic activity such as Na^+^-K^+^-ATPase, cPLA_2_, PI_3 _kinase and MAP kinase[9]. Otherwise, inhibition of PKC has been reported to prevent structure and function abnormalities in cardiomyopathy, heart failure, ischemic injury and so on[10]. Collectively, PKC activation is likely to be responsible for the pathology in diabetic cardiomyopathy, but the exact role that PKC plays in the alteration of cardiomyocytes cultured in high glucose levels and its underlying downstream signal transduction pathway is still not completely understood.

NF-κB is a transcription factor that directly regulates the expression of immediate-early genes and genes involved in the stress and inflammatory response following a variety of physiological or pathological stimuli[11,12]. Studies have found that activation of NF-κB may function as a causal event in the cardiac hypertrophic response of cardiomyopathy, as modeled in cultured cardiomyocytes and that NF-κB inhibition could attenuate or block the hypertrophy of cultured cardiomyocytes[13,14]. Recent studies have shown that oxidative stress generated by hyperglycemia is one of the major mediators of cardiac hypertrophy and dysfunction in diabetic cardiomyopathy, so NF-κB may function as a necessary mediator of the cardiac response in the pathogenesis of diabetic cardiomyopathy.

TNF-α is recognized as a significant contributor to myocardial dysfunction. Cardiomyocytes have been identified as a principal target of the proinflammatory actions of TNF-α. Significantly increased TNF-α expression is found in cardiac hypertrophy induced in stretched myocytes and in hemodynamic-over-loaded myocardium[15]. In heart failure, TNF-α transcription can be activated by NF-κB, and NF-κB itself is also dominantly regulated by TNF-α, as the increased expression of TNF-α triggers NF-κB translocation to the nucleus where it activates transcription of many inflammatory and immune response target genes.

c-fos is one of the immediate early genes and fetal contractile protein genes that regulates protein synthesis in cardiomyocytes. It is reported to be stimulated in ischemic injury, heart failure and cardiomyopathy[16]. What's more, increased expression of c-fos has also been reported in both Ang II-induced or mechanical stress-induced cardiomyocytes hypertrophy. PKC/c-fos pathway has been shown to be involved in endothelin-1-induced proliferation and hypertrophy of rat cardiac myocytes[17]. In this research, we used cultured neonatal ventricular myocytes as a model to study the influence of high glucose levels on the structure, function and expression of PKC, NF-κB, TNF-α and c-fos in cardiomyocytes, and tried to study the effect of PKC/NF-κB/c-fos signal transduction pathway in the pathogenesis of diabetic cardiomyopathy.

## Methods

### 1. Materials

#### 1.1 Experimental animals

1 day old Sprague-Dawley rats (provided by experimental animal center of Zhejiang University, Hangzhou, China), the investigation conforms with the Guide for the Care and Use of Laboratory Animals Published by the US National Institutes of Health (NIH Publication No. 85-23, revised 1996), and approval was granted by the university ethics review board, all procedures and materials prepared from animals were performed in accordance with institutional policies and guidelines.

#### 1.2 Drug and chemicals

Trypsin and DMEM were purchased from Gibico Chemical Co. Glucose, mannital, Ro-31-8220 and BAY11-7082 were obtained from Sigma Chemical Co. The concentrations of drugs used were based on previous studies. Primary antibodies included mouse anti PKC-α, rabbit anti PKC-β2, goat anti p-PKC-α, goat anti p-PKC-β2, mouse anti NF-κB, mouse anti p-NF-κB and rabbit anti c-fos antibody(Santa Cruz), Second antibodies included goat anti mouse, goat anti rabbit, rabbit anti goat antibody (Santa Cruz). The TNF-α primer (387 bp): 5'-GAA CAA CCC TAC GAG CAC CT-3'; 5'-GGG TAG TTT GGC TGG GAT AA-3'. dNTPs, Taq enzyme, M-MULV, MgCl_2_, RNasin were purchased from Promega Co; Oligo(dT) was obtained from Shanghai Sangon Biological Engineering Technology & Services Co.

### 2. Methods

#### 2.1 Culture of neonatal rat ventricular myocytes

1 day old Sprague-Dawley rats were sacrificed and their hearts were dissected. The ventricle was isolated, and digested with a buffer solution containing trypsin and collagenase for 20 min at 37°C. Ventricular myocytes were cultured described previously[18,19]. The cell supernatant was collected by centrifugation, after which pellet was resuspended and collected by centrifugation again, then resuspended with DMEM cell culture medium contain 15% fetal bovine serum. The above steps were repeated 3 to 5 times until the ventricles were completely digested. The cell suspension was then diluted to (1–5) × l0^5^/ml with DMEM cell culture medium contain 15% fetal bovine serum and penicillin-streptomycin and were placed in 6-well cell culture plates in humidified 5% CO_2_, 95% air atmosphere at 37°C. In the first three days, 5-Bru (0.1 mmol/L) was added to the culture medium to inhibit the growth of fibroblast[20]. Twenty-four hours after seeding, the culture medium was changed to serum-free DMEM and the cells were divided into different groups and cultured for another 48 h.

#### 2.2 Groups divided

After the initial culture, cells were divided into different glucose levels according to previously described researches as follow: control glucose (5 mmol/L) group, a high glucose (10 mmol/L, 15 mmol/L, 20 mmol/L, 25.5 mmol/L) group serials, equimolar mannital group (5 mmol/L glucose+20 mmol/L maninital); a high glucose add PKC inhibitor Ro-31-8220 (50 nmol/L) group and a high glucose add NF-κB inhibitor BAY11-7082 (5 μmol/L) group [21-23]. After the addition of those drugs, the cardiomyocytes in different groups were continued to be cultured for 48 h.

#### 2.3 Determination of cellular pulsatile frequency

Inverted microscope and dish were put into lucite couveuse, which equipped with homoiothermy air convection assembly. Make sure the dishes were covered fully by convection air at 37°C. For the study of cellular pulsatile frequency, five fields were randomly chosen from each group, and 20 individual cells were counted from each field at high power (×400)ocular lens of inverted microscope.

#### 2.4 Measurement of cell size

The volume of ventricular myocytes was obtained by measuring cellular diameter under an inverted microscope. For cell size measurements, five fields were randomly chosen from each group and 20 individual cells were counted from each field.

#### 2.5 Western blot analysis

The cells were washed twice with PBS and lysed at 4°C with lysis buffer, equal amounts of nuclear or total protein prepared from each lysate were resolved by SDS/PAGE [10%(w/v) gel] and transferred on PVDF membranes. After the membranes were washed with TBS (Tris-buffered solution) buffer to remove the stain, the filters were blocked with 5% nonfat milk in TBS for 1 hour at room temperature, and then incubated with diluted primary antibodies overnight at 4°C. After being washed three times with TBST (Tris-buffered solution containing 0.1% Tween 20) buffer for 10 min each, the filters were then incubation with horseradish peroxidase-conjugated second antibodies for 1 h, followed by washing three times with TBST buffer for 10 min each, at last the proteins were detected in the linear range of X-ray film by chemiluminescence reagent plus (ECL).

#### 2.6 RT-PCR analysis

The cultured cardiomyocytes were washed twice with PBS and lysed in 1 ml of Trizol reagent for 5 min. Total RNA was isolated with Trizol reagent according to the manufacturer's instruction. Total RNA was reversely transcribed and subsequently amplified by PCR using primers listed above. The resulting products were separated on 1.2% agarose gel and stained with ethidium bromide. The intensity of the bands was quantified by an image analysis scanning system, and normalized by GAPDH as control.

#### 2.7 Statistics

Each experiment shown was performed a minimum of three times, all data were presented as mean ± SE, and were analyzed by using SPSS 15.0 software. Comparisons among groups were made by an unpaired student's *t *test. A value of P < 0.05 was considered statistically significant.

## Results

### 3.1 Effect of different glucose levels on the pulsatile frequency and cellular size of cultured cardiomyocytes

After adding different treatment factors to different groups, and culturing for an additional 48 hours, the pulsatile frequency and cellular size of cardiomyocytes cultured in different glucose levels changed consistent with the increased glucose levels, but were unaffected by maninital as shown in Table 1. The cellular pulsatile frequency and diameters in different groups showed significant difference between each other, which all had statistical significance.

**Table 1:** Effect of high glucose on pulsatile frequency and cellular size of cultured cardiomyocytes

	**diameter(μm)**	**pulsatile frequency(bpm/min)**
Glucose (5 mmol/L)	18.76 ± 0.88	57.86 ± 2.103

Glucose (10 mmol/L)	19.98 ± 0.81*	59.08 ± 1.947*

Glucose (15 mmol/L)	20.71 ± 0.80*#	61.16 ± 2.121*#

Glucose (20 mmol/L)	21.33 ± 0.78* Δ	63.11 ± 2.269* Δ

Glucose (25.5 mmol/L)	25.33 ± 1.37* δ	70.43 ± 3.896* δ

iso-osmotic mannital	19.62 ± 1.43	57.85 ± 2.572

The pulsatile frequency and cellular size of cardiomyocytes cultured in different glucose levels increased consistent with the increased glucose levels as shown table 1. Treatment with mannital, used as an osmotic control, had no significant effect on the pulsatile frequency and cellular size of cardiomyocytes. The results were expressed as means ± SE (*n *= 100). * *P *< 0.05 vs control 5 mmol/L glucose group, #*P *< 0.05 vs 10 mmol/L glucose group, Δ*P *< 0.05 vs 15 mmol/L glucose group, δ*P *< 0.05 vs 20 mmol/L glucose group.

### 3.2 Effect of PKC inhibitor Ro-31-8220 and NF-κB inhibitor BAY11-7082 on the pulsatile frequency and cellular size of cardiomyocytes cultured in high glucose levels

The cardiomyocytes were divided into normal glucose (5 mmol/L), high glucose (25.5 mmol/L), high glucose adding Ro-31-8220 (50 nmol/L), high glucose adding BAY11-7082 (5 μmol/L), mannital (glucose 5 mmol/L+mannital 20.5 mmol/L), and cultured for an additional 48 hours. Compared with normal glucose group, the cellular pulsatile frequency and size of cardiomyocytes cultured in high glucose increased significantly, but not in iso-osmotic mannital group, after adding Ro-31-8220 and BAY11-7082, the pulsatile frequency and size of the cells all decreased significantly. The result was shown in Table 2.

**Table 2:** Effect of Ro-31-8220 and BAY11-7082 on pulsatile frequency and cellular size of cultured cardiomyocytes

	**diameter(μm)**	**pulsatile frequency(bpm/min)**
Normal Glucose (5 mmol/L)	18.76 ± 0.88	57.86 ± 2.103

High Glucose (25.5 mmol/L)	25.33 ± 1.37*	70.43 ± 3.896*

High Glucose +Ro-31-8220	21.41 ± 0.80#	64.73 ± 3.336#

High Glucose + BAY11-7082	20.94 ± 0.78#	62.55 ± 2.341#

iso-osmotic mannital	19.62 ± 1.43	57.85 ± 2.572

Compared with normal glucose(5 mmol/L) group, the cellular pulsatile frequency and size of cardiomyocytes cultured in high glucose(25.5 mmol/L) increased significantly, but not in iso-osmotic mannital group, after adding Ro-31-8220 and BAY11-7082, the pulsatile frequency and size of the cells all decreased significantly. The results were expressed as means ± SE (*n *= 100). * *P *< 0.05 vs control group(glucose:5 mmol/L), #*P *< 0.05 vs high glucose group (glucose: 25.5 mmol/L).

### 3.3 Effect of high glucose and PKC inhibitor Ro-31-8220 on the expression and activity of PKC in cardiomyocytes

**Figure 1:**
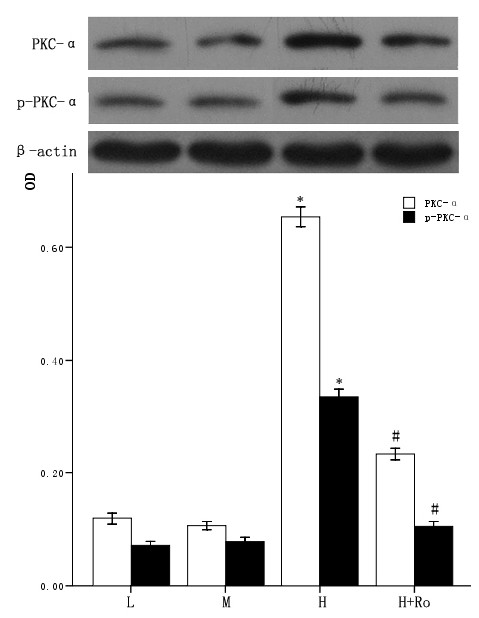
**Effect of high glucose levels and PKC inhibitor Ro-31-8220 on PKC-α expression in cultured cardiomyocytes**. Cardiomyocytes cultured in high glucose levels showed higher expression and activity of PKC-α compared with control group. After adding PKC inhibitor Ro-31-8220 (50 nmol/L) to the cardiomyocytes cultured in high glucose levels, the expression and activity of PKC-α decreased. Treatment with mannital, used as an osmotic control, had no significant effect on the expression of PKC-α in cardiomyocytes. The results were shown in figure1 expressed as means ± SE (*n *= 4 or 5). * *P *< 0.05 vs control group, #*P *< 0.05 vs high glucose group.

**Figure 2:**
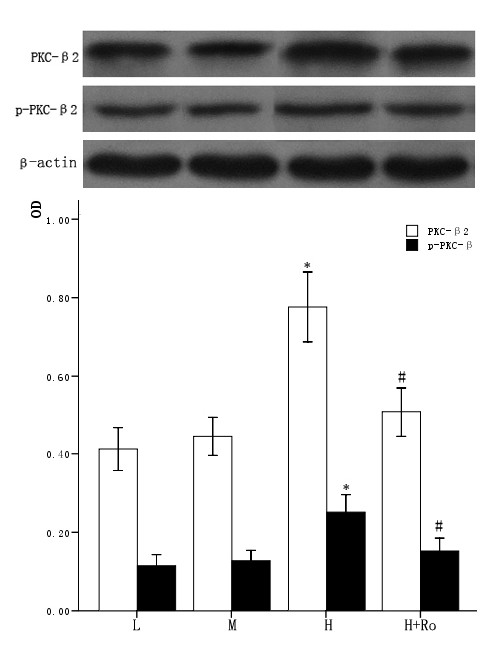
**Effect of high glucose levels and PKC inhibitor Ro-31-8220 on PKC-β2 expression in cultured cardiomyocytes**. Cardiomyocytes cultured in high glucose levels showed higher expression and activity of PKC-β2 compared with control group. After adding PKC inhibitor Ro-31-8220 (50 nmol/L) to the cardiomyocytes cultured in high glucose levels, the expression and activity of PKC-β2 decreased. Treatment with mannital, used as an osmotic control, had no significant effect on the expression of PKC-β2 in cardiomyocytes. The results were shown in figure 2 expressed as means ± SE (*n *= 4 or 5). * *P *< 0.05 vs control group, #*P *< 0.05 vs high glucose group.

The cardiomyocytes were divided into normal glucose (5 mmol/L), high glucose (25.5 mmol/L), high glucose adding Ro-31-8220 (50 nmol/L), mannital (glucose 5 mmol/L+mannital 20.5 mmol/L). After culturing for an additional 48 hours, the expression and activity of PKC-α and PKC-β2 were examined by western blot. Cardiomyocytes cultured in high glucose levels showed higher expression of PKC-α, PKC-β2 and increased activity of PKC-α, PKC-β2 at the same time, as reflected by higher expression of p-PKC-α, p-PKC-β2 compared with control group. After adding PKC inhibitor Ro-31-8220, the expression and activity of PKC-α, PKC-β2 decreased compared with the cardiomyocytes cultured in high glucose levels as shown in figure 1 and figure 2. Treatment with mannital, used as an osmotic control, had no significant effect on the expression of PKC-α and PKC-β2 in cardiomyocytes.

### 3.4 Effect of high glucose and PKC inhibitor Ro-31-8220, NF-κB inhibitor BAY11-7082 on the expression and activity of NF-κB in cardiomyocytes

**Figure 3:**
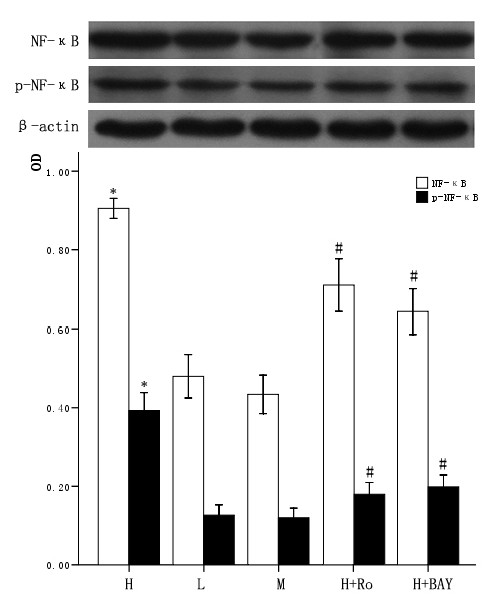
**Effect of high glucose and PKC inhibitor Ro-31-8220, NF-κB inhibitor BAY11-7082 on total cellular NF-κB expression in cardiomyocytes**. Cardiomyocytes cultured in high glucose levels showed higher expression and increased activity of NF-κB compared with control group. After adding PKC inhibitor Ro-31-8220(50 nmol/L) and NF-κB inhibitor BAY11-7082(5 μmol/L), the expression and activity of NF-κB decreased as shown in figure 3. Treatment with mannital, used as an osmotic control, had no significant effect on the expression of NF-κB in cardiomyocytes. The results were expressed as means ± SE (*n *= 4 or 5). * *P *< 0.05 vs control group, #*P *< 0.05 vs high glucose group.

**Figure 4:**
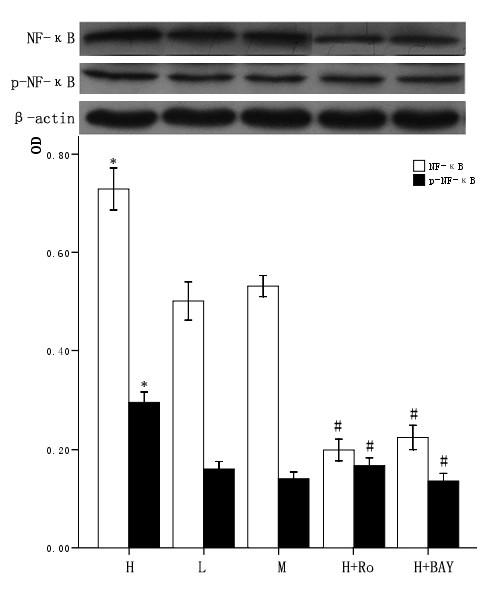
**Effect of high glucose and PKC inhibitor Ro-31-8220, NF-κB inhibitor BAY11-7082 on nuclear NF-κB expression in cardiomyocytes**. Cardiomyocytes cultured in high glucose levels showed higher nuclear protein expression and activity of NF-κB compared with control group. After adding PKC inhibitor Ro-31-8220(50 nmol/L) and NF-κB inhibitor BAY11-7082(5 μmol/L) the expression and activity of nuclear NF-κB decreased as shown in figure 4. Treatment with mannital, used as an osmotic control, had no significant effect on the nuclear expression of NF-κB in cardiomyocytes. The results were expressed as means ± SE (*n *= 4 or 5). * *P *< 0.05 vs control group, #*P *< 0.05 vs high glucose group.

The cardiomyocytes were divided into normal glucose (5 mmol/L), high glucose (25.5 mmol/L), high glucose adding Ro-31-8220 (50 nmol/L), high glucose adding BAY11-7082 (5 μmol/L), mannital (glucose 5 mmol/L+mannital 20.5 mmol/L). After culturing for an additional 48 hours, the expression of both total and nuclear NF-κB were examined by western blot. Total and nuclear expression of NF-κB, p-NF-κB all increased in cardiomyocytes cultured in high glucose levels compared with control group. After adding PKC inhibitor Ro-31-8220 and NF-κB inhibitor BAY11-7082, the whole or nuclear expression and activity of NF-κB decreased as shown in figure 3 and figure 4. Treatment with mannital, used as an osmotic control, had no significant effect on the expression of NF-κB in cardiomyocytes.

### 3.5 Effect of high glucose and PKC inhibitor Ro-31-8220, NF-κB inhibitor BAY11-7082 on the expression of TNF-α in cardiomyocytes

**Figure 5:**
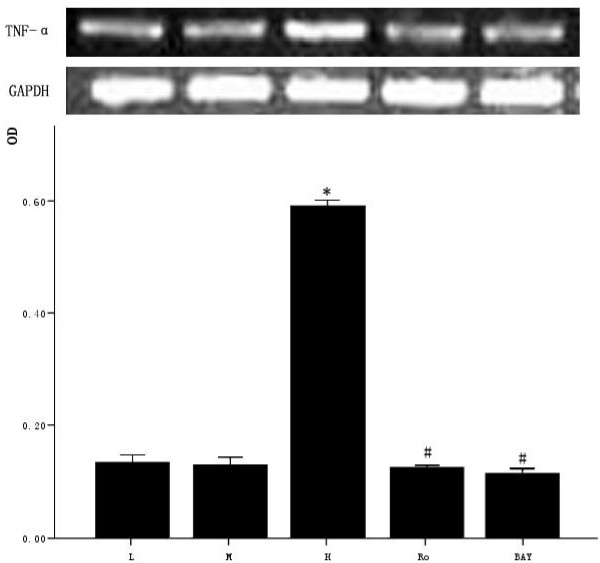
**Effect of high glucose and PKC inhibitor Ro-31-8220, NF-κB inhibitor BAY11-7082 on TNF-α expression in cardiomyocytes**. Cardiomyocytes cultured in high glucose levels showed higher expression of TNF-α compared with control group. After adding PKC inhibitor Ro-31-8220 (50 nmol/L) and NF-κB inhibitor BAY11-7082 (5 μmol/L), the expression of TNF-α decreased compared with the cardiomyocytes cultured in high glucose levels as shown in figure 5. Treatment with mannital, used as an osmotic control, had no significant effect on the expression of TNF-α in cardiomyocytes. The results were expressed as means ± SE (*n *= 4 or 5). * *P *< 0.05 vs control group, #*P *< 0.05 vs high glucose group.

The cardiomyocytes were divided into normal glucose (5 mmol/L), high glucose (25.5 mmol/L), high glucose adding Ro-31-8220 (50 nmol/L), high glucose adding BAY11-7082 (5 μmol/L), mannital (glucose 5 mmol/L+mannital 20.5 mmol/L). After culturing for an additional 48 hours, the expression of TNF-α was examined by RT-PCR. Cardiomyocytes cultured in high glucose levels showed higher expression of TNF-α compared with control group. After adding PKC inhibitor Ro-31-8220 and NF-κB inhibitor BAY11-7082, the expression of TNF-α decreased as shown in figure 5. Treatment with mannital, used as an osmotic control, had no significant effect on the expression of TNF-α in cardiomyocytes.

### 3.6 Effect of high glucose and PKC inhibitor Ro-31-8220, NF-κB inhibitor BAY11-7082 on the expression of c-fos in cardiomyocytes

**Figure 6:**
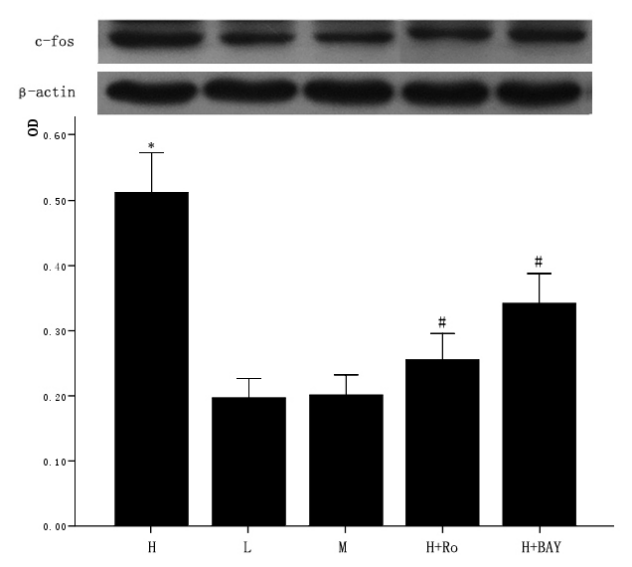
**Effect of high glucose and PKC inhibitor Ro-31-8220, NF-κB inhibitor BAY11-7082 on c-fos expression in cardiomyocytes**. Cardiomyocytes cultured in high glucose levels showed higher expression of c-fos compared with control group. After adding PKC inhibitor Ro-31-8220(50 nmol/L) and NF-κB inhibitor BAY11-7082(5 μmol/L) to the cardiomyocytes cultured in high glucose levels, the expression of c-fos decreased as shown in figure 6. Treatment with mannital, used as an osmotic control, had no significant effect on the expression of c-fos in cardiomyocytes. The results were expressed as means ± SE (*n *= 4 or 5). * *P *< 0.05 vs control group, #*P *< 0.05 vs high glucose group.

The cardiomyocytes were divided into normal glucose (5 mmol/L), high glucose (25.5 mmol/L), high glucose adding Ro-31-8220 (50 nmol/L), high glucose adding BAY11-7082 (5 μmol/L), mannital (glucose 5 mmol/L+mannital 20.5 mmol/L). After culturing for an additional 48 hours, the expression of c-fos was examined by western blot. Cardiomyocytes cultured in high glucose levels showed higher expression of c-fos compared with control group. After adding PKC inhibitor Ro-31-8220 and NF-κB inhibitor BAY11-7082, the expression of c-fos decreased as shown in figure 6. Treatment with mannital, used as an osmotic control, had no significant effect on the expression of c-fos in cardiomyocytes.

## Discussion

Diabetic cardiomyopathy is one of the most prevalent cardiovascular complications of diabetes mellitus, occurring independently of coronary artery disease and hypertension[2]. Its typically pathological changes include hypertrophy of cardiomyocytes, microangiopathy of heart and fibrosis of interstitial substance[24]. Cardiomyopathy in diabetic patients is characterized by early diastolic dysfunction, followed by late systolic dysfunction[25-27]. However, the mechanism underlying the development of structure and function impairment in cardiomyopathy remains unknown, in particular, the intracellular signal transduction pathway that leads to the pathological change of cardiomycyte hypertrophy is still unclear.

Our previous studies have shown that Sprague-Dawley rats with diabetic cardiomyopathy induced by alloxanm revealed a higher heart rate compared with control groups, and showed hypertrophy of cardiomyocytes, microangiopathy of heart, fibrosis of interstitial substance and subcellular remodeling in myocardium at the end 4, 6 weeks[28]. When examined by electron microscopy, it was characterized by damaged myofibrils and mitochondria, dilated and swollen sarcoplasmic reticulum[29]. There have been studies indicated that high glucose levels could increase the pulsatile frequency of cardiomyocytes as hyperglycemia regulates the opening of voltage-dependent channels existing in the cytoplasmic membrane due to activation of tyrosine kinase[30]. Accelerated pulsatile frequency could increase energy expenditure, aggravate the impairment of cardiomyocytes induced by high glucose levels, leading to energy insufficiency and a series of negative manifestations. In this study, we discovered that the cardiomyocytes cultured in high glucose levels showed increased pulsatile frequency and obvious hypertrophy compared with cells cultured in normal glucose levels, which conforms to our previous studies.

Protein kinase C has received increasing attention in the pathogenesis of cardiomyopathy as it has been shown to function as a target protein for second messengers. Over-expression of PKC-α and PKC-β2 were discovered in the early stage of pathological development of diabetic cardiomyopathy because sustained hyperglycemia in diabetes mellitus could activate DAG-PKC signal transduction pathway, leading to increased expression of PKC in cardiomyocytes and translocation of PKC from cytoplasm to cytomembrane[31-33]. The downstream signal transduction pathway, however, is still unclear. In our study, we investigated whether PKC was involved in the pathogenesis of diabetic cardiomyopathy, with particular focused on the role PKC plays in the onset of cardiomyocyte hypertrophy. In our research we found that the cardiomyocytes cultured in high glucose levels showed increased expression and activity of PKC-α, PKCβ2, as reflected by the higher protein expression of PKC-α, PKC-β2, p-PKC-α, p-PKC-β2 when examined by western blot, which was in agreement with the cellular defects induced in the cardiomyocytes by hyperglycemia. It is known that activation of PKC in cardiomyocyte could promote gene expression via many signal transduction pathways, such as MAPK (mitogen-activated protein kinase, MAPK), which can regulate the growth of cardiomyocytes. Other studies that done on ischemic and heart failure animal models have also shown that over-expression and activity of PKC could induce the expression of proto-oncogene such as c-fos, c-jun, leading to increased AP-1 (activator protein), with AP-1 potentially stimulate the transcription of type IV collagen, fibronectin, TGF-β and so on. In addition, many studies focused on heart failure have also discovered over-transcription of type IV collagen, fibronectin, TGF-β could induce impaired contractility of cardiomyocytes, increased accumulation of extracellular matrix and also hypertrophy of cardiomyocytes, microangiopathy of heart and fibrosis of interstitial substance, which at last leads to heart failure.

To further investigate and explain the mechanism of PKC on the high glucose induced cardiomyocyte impairment, we examined the expression of NF-κB, TNF-α and c-fos at the same time. We found that cardiomyocytes cultured in high glucose levels showed increased expression of NF-κB, TNF-α and c-fos compared with those cultured in the normal control glucose levels, and there was a significant difference existing between the two groups. NF-κB is a transcription factor that can directly regulate the expression of immediate-early genes and other genes involved in the stress response following a variety of physiological or pathological stimuli, and NF-κB inhibition could attenuate or block the hypertrophic response of cultured cardiomyocytes[34]. Recent studies have shown that oxidative stress generated by hyperglycemia is one of the major mediators of diabetic cardiomyopathy, and activation of NF-κB is a possible mechanism for oxidative stress-induced cardiovascular complications in diabetes. On the assumption that NF-κB activated by PKC may function as a core effect in the cardiac hypertrophic response of diabetic cardiomyopathy, as modeled in cultured cardiomyocytes, we used PKCα/β2 inhibitor (Ro-31-8220) and NF-κB inhibitor (BAY11-7082) to study the signal transduction pathway of PKC and its downstream effect on NF-κB. We discovered that after using the selective PKCα/β2 inhibitor Ro-31-8220, the hypertrophy of cardiomyocytes induced by high glucose concentration was obviously reversed, with the depressed expression of both PKC-α, PKC-β2 as well as NF-κB, TNF-α and c-fos. Similarly, after using the NF-κB inhibitor BAY11-7082, both the cardiac hypertrophy and the increased expression of NF-κB, TNF-α, c-fos were reversed remarkably. For this phenomenon, we supposed that hyperglycemia could result in a higher content of intracellular DAG in the cardiomyocytes, leading to increased expression and activity of PKC-α, PKC-β2, then inducing over-expression of NF-κB via PKC-IKK-NF-κB signal transduction pathway[35]. It is known that after entering cellular nucleus, NF-κB could increase the transcription of a number of inflammatory cytokines, including TNF-α, which is one of the relevant mediators of cardiomyopathy disease states[36-39]. For example, TNF-α was proved to modulate heart failure by provoking hypertrophic growth in cardiac myocytes, and a recent study in neonatal cardiac myocytes showed that TNF was sufficient to trigger cardiac myocyte hypertrophy. So TNF-α was considered as parameters of hypertrophy that could be induced by angiotension II, endothelin-1, phenylephrine, myotrophin and so on, but it could be obviously inhibited through over-expression of degradation resistant mutant of IêBα, which support the hypothesis that increased NF-κB expression and consequently activation of TNF-α is involved in the cardiomyocyte hypertrophic response induced by high glucose levels[40,41]. Studies have demonstrated that the increased expression of TNF-α could further lead to the expression of proto-oncogene, such as c-fos, which would result in an accelerated rate of general protein synthesis, as well as an increase in LV mass caused by increased cell size and protein content of cardiomyocytes. Increased expression of c-fos was viewed as an adaptive response for maintaining cardiac output after stimulation of with TNF or AngII, as in isolated adult cardiac myocytes or heart failure, ischemic injury and stress-induced hypertrophy models. Those pathological changes ultimately result in remodeling of the cardiac muscle, leading to development of diabetic cardiomyopathy.

In conclusion, our study indicated for the first time that high glucose levels can significantly influence the structure and function of cultured cardiomyocytes, causing cardiac hypertrophy that may occur via PKC/NF-κB/c-fos pathway, which leads to diabetic cardiomyopathy.

## Conclusion

Our study indicated for the first time that high glucose levels can significantly influence the structure and function of cultured cardiomyocytes, causing cardiac hypertrophy via PKC signal transduction pathway, which may lead to diabetic cardiomyopathy.

## Abbreviations

PKC: Protein Kinase C; NF-κB: Nuclear factor-κB; TNF-α: tumor necrosis factor-α; DAG: diacylglycerol; PLA: phospholipase a; PI: phosphatidyl inositol; MAP: mitogen-activated protein; TBS: Tris-buffered solution; TBST: Tris-buffered solution containing 0.1%(v/v) Tween 20; ECL: enhanced chemiluminescence; MAPK: mitogen-activated protein kinase; AP-1: activator protein-1; TGF-β: transforming growth factor-β; IKK: I-κB kinase; IêBα: inhibitory protein α of NF-κB.

## Competing interests

The authors declare that they have no competing interests.

## Authors' contributions

MW carried out the molecular studies, participated in the sequence alignment and drafted the manuscript. WBZ participated in the design of the study and performed the statistical analysis. GSF and BQZ conceived of the study, and participated in its design and coordination. JHZ helped to draft the manuscript. All authors read and approved the final manuscript.

## Author information

All authors' affiliations are the Department of Cardiovascular Diseases, Sir Run Run Shaw Hospital, College of Medicine, Zhejiang University. Professor Fu Guosheng is the president of the Department of Cardiovascular Diseases, Sir Run Run Shaw Hospital, Zhou Binquan is an attending doctor in our department, Zhu Junhui and Zhang Wenbin are the fellow doctors in our department, Wang Min is a medical doctor in College of Medicine, Zhejiang University.
